# The efficacy and safety of continuous theta burst stimulation for auditory hallucinations: a systematic review and meta-analysis of randomized controlled trials

**DOI:** 10.3389/fpsyt.2024.1446849

**Published:** 2024-08-19

**Authors:** Shi-Yi Ye, Chun-Nuan Chen, Bo Wei, Jin-Qiong Zhan, Yi-Heng Li, Chen Zhang, Jing-Jing Huang, Yuan-Jian Yang

**Affiliations:** ^1^ Department of Psychiatry and Biological Psychiatry Laboratory, Jiangxi Mental Hospital & Affiliated Mental Hospital, Jiangxi Medical College, Nanchang University, Nanchang, China; ^2^ The 3^rd^ Clinical Medical College, Jiangxi Medical College, Nanchang University, Nanchang, China; ^3^ Department of Neurology, The Second Clinical Medical College, The Second Affiliated Hospital, Fujian Medical University, Quanzhou, China; ^4^ Nanchang City Key Laboratory of Biological Psychiatry, Jiangxi Provincial Clinical Research Center on Mental Disorders, Jiangxi Mental Hospital, Nanchang, China; ^5^ Shanghai Mental Health Center, Shanghai Jiao Tong University School of Medicine, Shanghai, China; ^6^ Shanghai Pudong New Area Mental Health Center, School of Medicine, Tongji University, Shanghai, China

**Keywords:** continuous theta burst stimulation (cTBS), auditory hallucinations, treatment, meta-analysis, non-invasive brain stimulation

## Abstract

**Objective:**

Auditory hallucinations are the most frequently occurring psychotic symptom in schizophrenia. Continuous theta burst stimulation (cTBS) has been used as an adjuvant treatment for auditory hallucinations. This meta-analysis focused on randomized controlled clinical trials (RCTs) to assess the efficacy of adjuvant cTBS on auditory hallucinations in schizophrenia.

**Methods:**

We performed a comprehensive search of four international databases from their inception to January 14, 2024, to identify relevant RCTs that assessed the effects of adjuvant cTBS on auditory hallucinations. The key words included “auditory hallucinations”, “continuous theta burst stimulation” and “transcranial magnetic stimulation”. Inclusion criteria included patients with auditory hallucinations in schizophrenia or schizoaffective disorder. The Revised Cochrane risk-of-bias tool for randomized trials (RoB1) were used to evaluate the risk of bias and the Review Manager Software Version 5.4 was employed to pool the data.

**Results:**

A total of 4 RCTs involving 151 patients with auditory hallucinations were included in the analysis. The Cochrane risk of bias of these studies presented “low risk” in all items. Preliminary analysis showed no significant advantage of adjuvant cTBS over sham stimulation in reducing hallucinations [4 RCTs, n = 151; SMD: -0.45 (95%CI: -1.01, 0.12), *P* = 0.13; I^2^ = 61%]. Subgroup analysis revealed that patients treated with adjuvant cTBS for more than 10 stimulation sessions and total number of pulses more than 6000 [3 RCTs, n = 87; SMD: -4.43 (95%CI: -8.22, -0.63), *P* = 0.02; I^2^ = 47%] had a statistically significant improvement in hallucination symptoms. Moreover, the rates of adverse events and discontinuation did not show any significant difference between the cTBS and sham group.

**Conclusions:**

Although preliminary analysis did not revealed a significant advantage of adjuvant cTBS over sham stimulation, subgroup analysis showed that specific parameters of cTBS appear to be effective in the treatment of auditory hallucinations in schizophrenia. Further large-scale studies are needed to determine the standard protocol of cTBS for treating auditory hallucinations.

**Systematic review registration:**

https://www.crd.york.ac.uk/prospero/, identifier CRD42024534045.

## 1 Introduction

Auditory hallucination refers to the perception of another person’s voice in the absence of any external stimuli (1), usually occurring in individuals with mental illness. It is estimated that approximately 60-80% of schizophrenia patients (2) and 40% of major depression patients experience auditory hallucinations (3, 4). These hallucinations can be extremely distressing and painful, particularly when they are abusive, demeaning, or commanding in nature (5). Chronic hallucinations would impair patients’ emotional perception and increase the risk of suicidal behaviors or violence (6, 7). Although current antipsychotic medications are effective in managing auditory hallucinatory symptoms, up to 30% of the patients cannot receive any relief (8).

Brain connectivity dysfunction is thought to be the basis of auditory hallucinations (9). In 1998, Karl J proposed that alterations in prefrontal and temporal cortex connectivity were responsible for hallucinations (10). Subsequent studies, using brain imaging techniques, showed that there was increased activity in the frontotemporal lobe regions of patients with auditory hallucinations (11, 12). Abnormal connectivity between the auditory cortex and other cortical regions was also found in patients with auditory verbal hallucinations (13, 14). Based on the ‘brain connectivity dysfunction’ hypothesis, nonpharmacological treatment strategies that regulate brain activity, such as electroconvulsive therapy (ECT) (15) and non-invasive brain stimulation techniques (16), have been introduced to increase the therapeutic efficiency of antipsychotics (APs) on hallucinations.

Repetitive transcranial magnetic stimulation (rTMS) is a non-invasive neuromodulation technique that has been widely recommended for the treatment of neuropsychiatric disorders (17–19). Studies have shown that rTMS can alleviate auditory hallucinations by influencing cortical excitability, and its therapeutic effect is affected by the frequency and site of stimulation (20, 21). Normally, low frequency rTMS (LF; < 5Hz) results in persistent alterations in the inhibitory activity of target regions, whereas high frequency rTMS (HF; ≥ 5Hz) tends to have an excitatory effect (22). The temporo-parietal junction (TPJ) is a connection of the primary (PAC) and secondary (SAC) auditory cortices and showed overactivity during episodes of auditory verbal hallucinations (23). Low-frequency rTMS can reduce the intensity of auditory hallucinations by reducing the activity of TPJ (24). Hoffman et al. also reported a decrease in the intensity of auditory hallucinations after applying 1 Hz TMS to the left TPJ (25). However, despite the initial enthusiasm generated by positive studies, the efficacy of inhibitory rTMS in the treatment of auditory hallucinations in schizophrenia remains controversial. The latest meta-analysis on the subject, by Guttesen et al. (26), did not show superiority over placebo. Furthermore, the conventional rTMS protocols still have certain practical limitations, such as modest and short-lasting effects, and a delayed time-to-response. To enhance the therapeutic effects of rTMS, novel types of rTMS such as theta burst stimulation (TBS) have been developed.

TBS is a stimulation pattern that imitates the natural activity of the brain during learning tasks (27). It delivers three biphasic pulses at a frequency of 50 Hz in bursts separated by 200 ms at a frequency of 5 Hz. This stimulation protocol has several advantages over traditional TMS. It offers a longer-lasting ability to modulate cortical excitability, shorter stimulation session, and greater efficacy at lower stimulation intensities (28, 29). Continuous TBS (cTBS), a form of TBS that produces 20 minutes of suppression with 20 seconds of stimulation, is a quicker and potentially more effective technique to reduce cortical hyperactivity. Preliminary findings have suggested that cTBS therapy may yield positive results in improving auditory hallucination and minimizing adverse consequences (30–33). Among them, one randomized controlled trials (RCTs) of 50 patients showed that active cTBS treatment exhibited an odds ratio of 5.6 for auditory hallucinations response compared with sham stimulation (32). However, Koops S et al. reported that adjuvant cTBS treatment in patients with auditory hallucinations did not show an advantage over sham stimulation (34). Given the results of studies on the efficacy of cTBS are controversial, we here conducted a meta-analysis of RCTs of adjuvant cTBS in patients with auditory hallucinations, aiming to assess the clinical efficacy and safety of adjuvant cTBS in the treatment of auditory hallucinations.

## 2 Method

This systematic review was registered with PROSPERO (CRD42024534045) [see **Supplementary Materials** for the PROSPERO register].

### 2.1 Eligibility criteria

This meta-analysis followed the Preferred Reporting Items for Systematic Reviews and Meta-analyses (PRISMA) guidelines (35) and utilized the PICOS acronym to determine inclusion criteria. The participants were patients with auditory hallucinations in schizophrenia or schizoaffective disorder, and the intervention involved active cTBS plus APs versus sham or placebo stimulation plus APs. The included subjects did not respond sufficiently to at least one antipsychotic medication administered for a minimum of 6 weeks or longer at the highest acceptable dosage. During cTBS and sham stimulation therapy, patients received the same dose and type of antipsychotic medication as before treatment. The primary outcome measure is the improvement in auditory hallucination symptoms as assessed by standardized scales such as the Auditory Hallucination Rating Scale (AHRS) or Psychotic Symptom Rating Scales-Auditory Hallucination (PSYRST-AH) at the end of cTBS treatment. Secondary outcomes include the rate of adverse events as measured by the Global Index of Safety (GIS), changes in other psychotic symptoms as assessed by the Positive and Negative Symptom Scale (PANSS), and discontinuation due to any reason. This study included only RCTs evaluating the efficacy and safety of cTBS in the treatment of auditory hallucinations, and did not include animal studies, observational studies, and review articles on cTBS in the treatment of auditory hallucinations. When there was overlapping data across multiple published articles, only articles with complete data were included in the analysis.

### 2.2 Search strategy

Two researchers independently searched for relevant RCTs across four major international databases, including PubMed, EMBASE, Web of Science, and the Cochrane Library, from their inception dates up to January 14, 2024. The search terms used were (Transcranial Magnetic Stimulation OR continuous theta burst stimulation OR theta burst stimulation OR theta-burst stimulations OR theta burst transcranial magnetic stimulation OR transcranial theta burst stimulation OR TBS OR cTBS) AND (hallucinations OR Auditory Hallucination, Verbal) OR (Verbal Auditory Hallucination OR Verbal Auditory Hallucinations OR Auditory Hallucinations, Verbal OR Auditory Hallucination OR Auditory Hallucinations OR Phonism OR Voice).

### 2.3 Data extraction

Two investigators (Ye S-Y, Chen C-N) collaborated in the study selection and data extraction procedures. They employed a predetermined form to gather pertinent information, and in the event of any discrepancies, they resolved them through discussions and consultation with a senior researcher (Wei B). Furthermore, the first and/or corresponding authors were contacted when deemed necessary to obtain clarification or missing data.

### 2.4 RoB of included studies and certainty of overall evidence

Two researchers (Zhan J-Q, Li Y-H) individually evaluated the quality of all the studies included using the Cochrane Collaboration’s bias risk assessment tool (ROB1.0) (36). This tool examines five areas, such as randomization process, deviations from intended interventions, missing outcome data, measurement of the outcome, and selection of the reported result. The tool assigns a risk of bias as “high risk”, “low risk”, or “some concerns” for each study. In case of any disagreements, the entire review team discussed and resolved the issue. Similarly, the two examiners (Zhan J-Q, Li Y-H), carried out an independent evaluation of the overall strength of evidence for each meta-analytic outcome using the GRADE system (37, 38).

### 2.5 Data analysis

In this meta-analysis, we employed the Review Manager Software Version 5.4 (Cochrane IMS, Oxford, United Kingdom) to examine the efficacy and safety of cTBS treatment. To analyze continuous outcomes, we calculated standardized mean differences (SMDs) with 95% confidence intervals (CIs), while risk ratios (RRs) with 95% CIs were used for dichotomous data. Following the recommendation of previous study (39), we utilized the random-effects model to generate all meta-analytic outcomes. We evaluated heterogeneity among studies using Cochrane’s Q and I^2^ test. If the *P*-value was less than 0.1 and the I^2^-value was greater than 50%, it indicated significant study heterogeneity (40). The publication bias was assessed through visual funnel plot inspection and the Egger test (41), if there were at least 10 eligible RCTs in the meta-analysis (42). As the result of preliminary analysis was negative, we did not further calculate the failed-safe number. We established a significance level of *P* < 0.05 for this meta-analysis.

## 3 Results

### 3.1 Literature search and study selection

**Figure 1:**
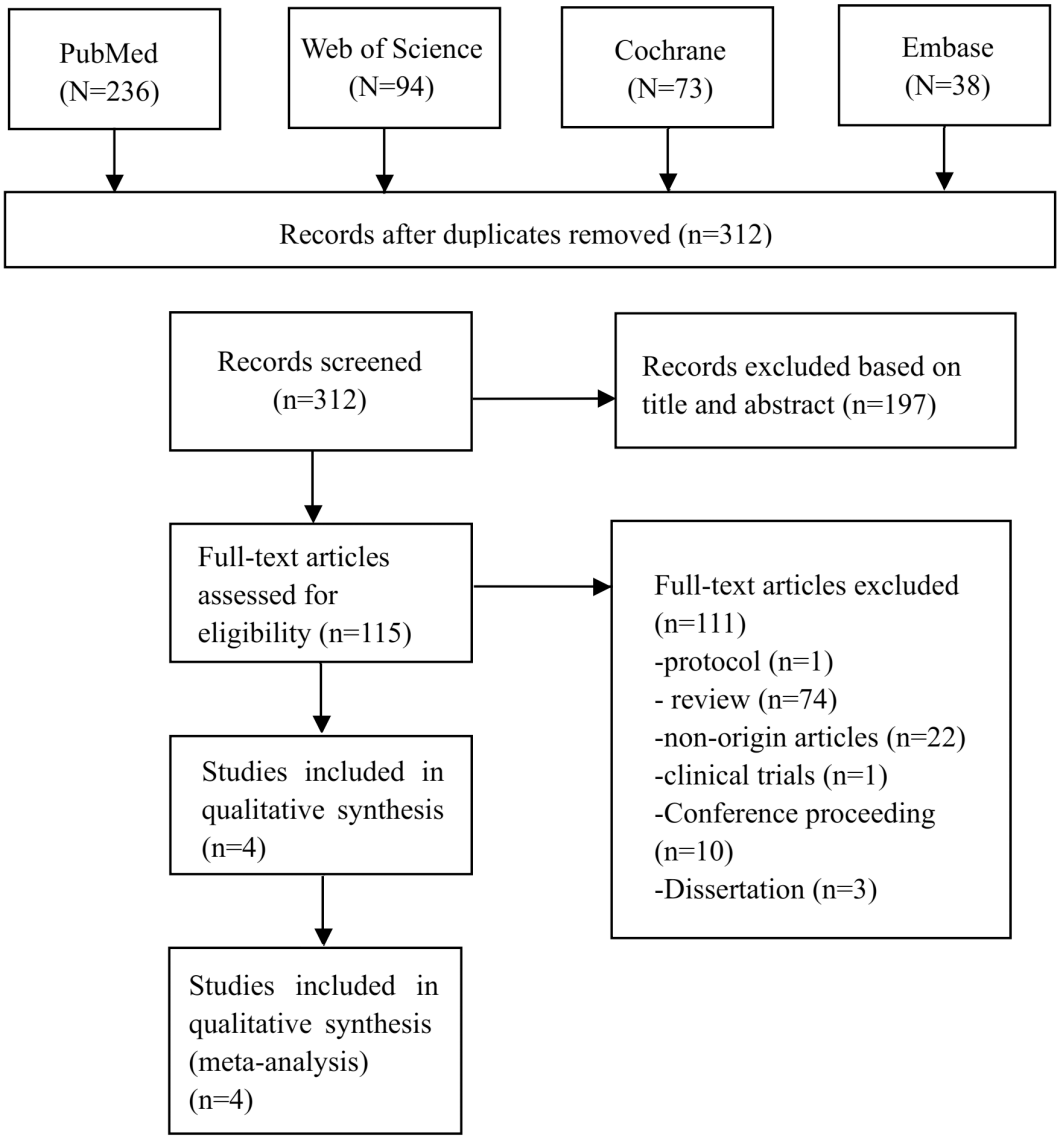
PRISMA flow diagram.

As depicted in **Figure 1**, a total of 441 publications were sourced from English databases. After applying the inclusion criteria, 4 research trials (n = 151) (30–32, 34) were identified as eligible for the meta-analysis.

### 3.2 Study characteristics

The **Table 1** provides an overview of the features of the RCTs that were included in the meta-analysis. Four RCTs were performed to compare the efficiency of active cTBS plus APs (n = 75) and sham cTBS plus APs (n = 76). The average age of patients was 37.78 (standard deviation, SD = 13.51). These four RCTs were conducted in different countries, with one each in China (n = 22), Germany (n = 15), India (n = 50) and Netherlands (n = 64). The duration of cTBS treatment ranged from 1 to 3 weeks, and the treatment sessions ranged from 10 to 80. The magnetic stimulus targeted the temporoparietal cortex.

**Table 1:** Participants characteristics and cTBS parameters of the included studies.

Study (research site)	Number of patients	Mean age (years)		cTBS treatment during weeks	Intervention versus control groups; number of patients (n)	-Site -Intensity -Frequency (HZ)	-Pulses per session -Number of sessions -Total pluses
		Active cTBS	Sham cTBS				
Plewnia et al., 2014 (31) (Germany)	15	45.6 ± 6.0	48.3 ± 17.4	3	1. Active cTBS + APs; n=7 2. Sham cTBS + APs; n=8	TPC -80%RMT -50	-600 -15 -9000
Koops et al., 2016 (34) (Netherlands)	64	38.0 ± 15.0	42.0 ± 13.0	1	1. Active cTBS + APs; n=32 2. Sham cTBS + APs; n=32	LTPC -80%AMT -50	- 600 -10 -6000
Tyagi et al., 2022 (32) (India)	50	32.17 ± 11.22	33.31 ± 11.48	2	1. Active cTBS + APs; n=25 2. Sham cTBS + APs; n=25	LTPC and RTPC -80%RMT -50	-1200 -20 -24,000
Liu et al., 2023 (30) (China)	22	38.27 ± 12.67	37.27 ± 10.55	2	1. Active cTBS + APs; n=11 2. Sham cTBS + APs; n=11	LTPJ -80%RMT -50	-1800 -80 -144,000

cTBS, continuous theta burst stimulation; APs, antipsychotics; PC, temporoparietal cortices; LTPC, left temporoparietal cortices; RTPC, right temporoparietal cortices; LTPJ, left temporo-parietal junction; AMT, active motor threshold; RMT, resting motor threshold.

### 3.3 RoB of included studies and certainty overall evidence

**Supplementary Table 1** summarize the Cochrane risk of bias. The random sequence generation and allocation concealment, performance bias, attrition bias and reporting bias in all four RCTs (4/4, 100%) were rated at “low risk”. Additionally, the overall evidence level for the four meta-analytic outcomes was rated as moderate (100%, 4/4) according to the GRADE approach, as shown in **Supplementary Table 2**.

### 3.4 The improvement of auditory hallucination symptoms

**Figure 2:**
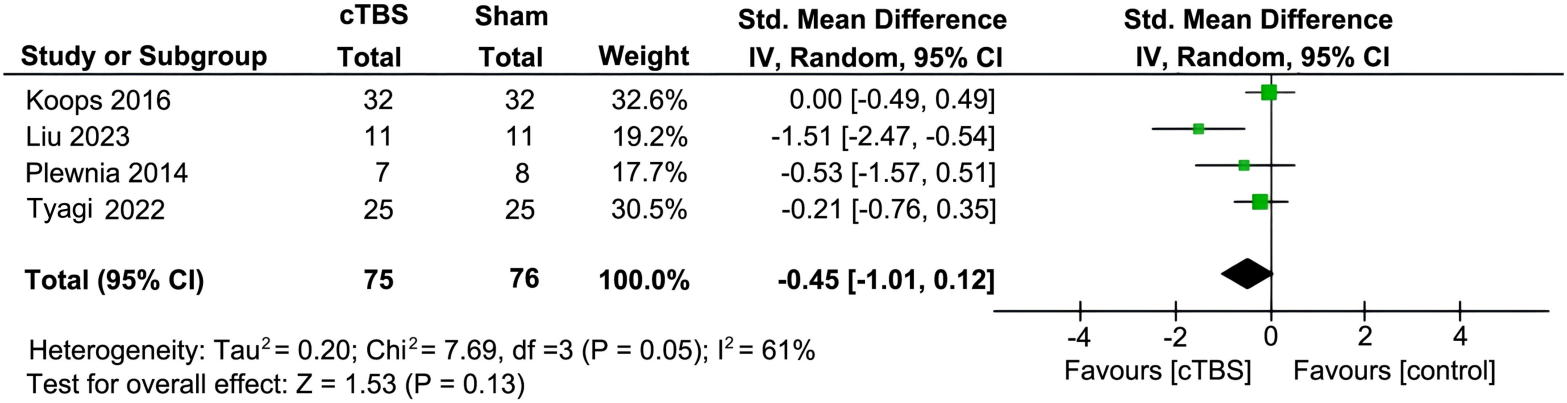
Forest plot for auditory hallucinations as measured by the Auditory Hallucination Rating Scale (AHRS) and the Psychotic Symptom Rating Scales-Auditory Hallucination (PSYRST-AH) post cTBS treatment.

Preliminary analysis showed that in four RCTs involving 151 participants, there was no significant difference in improvement of auditory hallucination symptoms between the active cTBS and the sham group, as assessed by AHRS and PSYRAT-AH [SMD = -0.45 (95% CI: -1.01, 0.12), I^2 = ^61%; *P* = 0.13; **Figure 2**]. After one study with an outlier effect size was removed, the statistical analysis remained stable. However, subgroup analyses found that when stimulation session > 10 and the total number of pulses > 6000 [3 RCTs, n = 87; SMD: -4.43 (95%CI: -8.22, -0.63), *P* = 0.02; I^2^ = 47%], active cTBS produced a significant improvement of auditory hallucination symptoms in patients as compared to sham stimulation (**Table 2**). The frequency of cTBS stimulation, whether 2 sessions/day or more, did not show an advantage over the sham group (*P* > 0.05).

**Table 2:** Subgroup analyses.

Variables		Studies (subjects)	SMDs (95%CI)	I^2^ (%)	*P*-value
**Stimulation sessions**	≤10	1 (64)	0.00 (-0.49, 0.49)	NA	1.00
**Total number of pulses**	>6000	3 (87)	-4.43 (-8.22, -0.63)	47	**0.02**
	≤6000	1 (64)	0.00 (-0.49, 0.49)	NA	1.00

Bolded P-values: P < 0.05.

CI, confidence interval; SMDs, standard mean differences; NA, not available.

### 3.5 Psychotic symptoms and discontinuation

Although adjuvant cTBS treatment alleviated the auditory hallucinations of patients, it did not improve the total psychopathological symptoms compared to the sham group, as measured by the Positive and Negative Symptom Scale (PANSS-P/N/G) [PANSS-P: 3 RCTs, n = 134, SMD = -1.08 (95% CI: -4.52, 0.91), *P* = 0.19; I^2^ = 64%; PANSS-N: 2 RCTs, n = 72; SMD = -0.36 (95% CI: -3.01, 2.29), *P* = 0.79; I^2^ = 0%; PANSS-G: 2 RCTs, n = 72; SMD = -0.27 (95% CI: -3.34, 2.8), *P* = 0.86; I^2^ = 7%] (**Table 3**).

**Table 3:** Secondary outcomes: adjunctive cTBS for patients with auditory hallucinations.

Variables	Studies (subjects)	SMDs/RRs (95%CI)	I^2^ (%)	*P*-value
Adverse event				
pians	2 (80)	1.19 (0.73, 1.95)	0	0.48
headache	2 (66)	1.20 (0.41, 3.54)	0	0.74
**Discontinuation due to any reason**	3 (146)	1.68 (0.68, 4.16)	0	0.26
PANSS scores				
PANSS-NS	2 (72)	-0.36 (-3.01, 2.29)	0	0.79
PANSS-PS	3 (134)	-1.08 (-4.52, 0.91)	64	0.19
PANSS-GS	2 (72)	-0.27 (-3.34, 2.8)	7	0.86

PANSS-NS, Positive and Negative Syndrome Scale; Negative scale score.

PANSS-PS, Positive and Negative Syndrome Scale; Positive scale score.

PANSS-GS, Positive and Negative Syndrome Scale; General Psychopathology scale score.

CI, confidence interval; SMDs, standard mean differences; RRs, relative risks.

Only three RCTs reported adverse events. There was no significant difference between the cTBS and the sham group regarding the occurrence of pians [2 RCTs, n = 80; RR = 1.19 (95% CI: 0.73, 1.95), *P* = 0.48; I^2^ = 0%] and headaches [2 RCTs, n = 66; RR = 1.20 (95% CI: 0.41, 3.54), *P* = 0.74; I^2^ = 0%] (**Table 3**). Similarly, no difference between the active cTBS and the sham group was observed regarding discontinuation for any reason [3 RCTs, n = 146; RR = 1.68 (95% CI: 0.68, 4.16), *P* = 0.26; I^2^ = 0%] (**Table 3**).

### 3.6 Publication bias

Four RCTs were included in this meta-analysis, however, we were unable to meet the criteria for the Egger test, which requires a minimum of ten studies. As a result, we did not evaluate the potential for publication bias for the primary outcome in this analysis.

## 4 Discussion

To the best of our knowledge, this is the first meta-analysis to evaluate the efficacy and safety of cTBS in the treatment of auditory hallucinations. We show that adjuvant cTBS treatment with stimulation sessions > 10 and a total number of pluses > 6000 could significantly improve auditory hallucination symptoms in patients. In addition, cTBS treatment did not increase adverse events and discontinuation to any reason, suggesting that cTBS is safe and well-tolerated for treating auditory hallucinations.

As a form of rTMS, TBS has been utilized to modulate neural network abnormalities associated with neuropsychiatric disorders (43, 44). TBS can be delivered either continuously (cTBS), which has an inhibitory effect, or intermittently (iTBS) in 2-second trains separated by 8-second intervals, which is considered excitatory (28). Previous study has shown that LF-rTMS targeting TPJ, a brain region closely associated with auditory hallucinations, can reduce the intensity of auditory hallucinations in individuals experiencing psychotic symptoms (25). Therefore, cTBS therapy targeting TPJ is theoretically also effective for auditory hallucinations. Four RCTs have been conducted to study the efficacy and safety of adjuvant cTBS treatment for auditory hallucinations. Meta-analysis of these RCTs revealed that there was no significant advantage of active cTBS over sham in reducing auditory hallucination symptoms in the total of 151 participants. Considering that the effectiveness of TBS depends on various parameters, such as stimulation time per session, stimulus intensity, stimulation session, and stimulus site, thus we further analyzed the efficacy of cTBS for the treatment of auditory hallucinations under different conditions. We found that when stimulation session > 10 and a total number of pluses > 6000, cTBS treatment could significantly improve the auditory hallucination symptoms in patients. This finding demonstrates that appropriate parameters determine the clinical efficacy of cTBS for the treatment of auditory hallucinations.

Increased pulse number of TBS can have a greater cumulative effect on cortical excitability and functional connectivity (45). A greater number of sessions and higher total pulse dose appear to produce superior clinical efficacy (46, 47). Furthermore, it has been suggested that amelioration of auditory hallucinations might only occur gradually after prolonged treatment (48). One of these RCTs studied in the meat-analysis, in which the participants received TB-rTMS or placebo treatment twice a day for 5 consecutive days (10 sessions), showed that improvement of auditory verbal hallucinations did not differ significantly between the TB-rTMS and the placebo group as measured with both the PSYRST-AH and the AHRS (34). We postulate that the negative results in this RCT might be attributable to the limited treatment session and period. Compared to this study (34), the other three studies administered more treatment sessions over a more extended period (30–32). Specifically, Liu et al., adopted a high-dose accelerated 1800-pulse cTBS (cTBS_1800_) protocol, which consists of a total of 80 cTBS_1800_ sessions during two consecutive weeks (5 days on, 2 days off), and found a significant difference between the active cTBS and sham group in terms of improvement of auditory hallucinations (30). An increase in the number and intensity of cTBS has also been shown to improve treatment efficacy in clinical trials of major depressive disorder, where the dose-effect of cTBS has been identified (49). Therefore, it is necessary to explore appropriate cTBS stimulation parameters to ensure that it is safe and well tolerated in patients with auditory hallucinations and has therapeutic properties in the future.

It should be noted that the included studies targeted the left (and right for one study) temporo-parietal junction, based simply on the median zone between T3 and P3 of the 10-20 EEG system. This approach has been criticized for not accounting for inter-individual variability and the variability of the zones whose hyperactivity is associated with hallucinations. It has been suggested that the use of neuronavigation based on structural or functional targets would be more effective (50, 51). However, the subgroup analysis of this meta-analysis found that cTBS targeting temporo-parietal junction based on the median zone between T3 and P3 of the EEG system with specific parameters has a superior therapeutic effect than sham stimulation, indicating that neuronavigation-based targeting approaches may not be necessary for cTBS therapy. Of course, if the conditions are available, the use of neuronavigation technology will be more conducive to the efficacy of TBS in the treatment of auditory hallucinations.

The AHRS has perfect psychometric properties for detecting the changes in auditory hallucinations, and is therefore recommended for the measurement of auditory hallucination symptoms in schizophrenia (52). Other scales, such P3 item in the PANSS and PSYRST-AH, are also used to measure auditory hallucinations, but they appear to be less reliable for detecting the changes in hallucination symptoms. Previous studies have also reported that the use of different scales can lead to differences in measures of auditory hallucinations symptoms (53). In this meta-analysis, only 2 RCTs (32, 34) adopted AHRS to evaluate auditory hallucinations, thus the standardized data might be biased against primary outcomes. Hence, it is suggested that future studies should consistently use AHRS to obtain more credible and reliable results.

It is imperative to note several limitations in the present meta-analysis. Firstly, all four RCTs included had relatively small sample sizes, which limited the statistical power of the study. Secondly, the concomitant use of antipsychotics was neither standardized nor consistently reported in the included studies, which precludes the exploration of potential confounding effects of clinical medications. Thirdly, some secondary outcomes were analyzed based on a small number of studies, which also reduced the likelihood of detecting significant findings. Lastly, the current literature search strategy may have selection bias since the search was limited to English-language database.

In this meta-analysis, we show that adjuvant cTBS treatment can produce a therapeutic effect on auditory hallucinations and this treatment appears to be safe and well-tolerated in patients. Given the important impact of different parameters such as stimulation session and number of pulses on the efficacy of cTBS, multicenter RCTs with a larger sample size are needed to determine an appropriate and standard cTBS protocol for the treatment of auditory hallucinations in the future.

## Data Availability

The original contributions presented in the study are included in the article/**Supplementary Material**. Further inquiries can be directed to the corresponding authors.
