# Supplementary material for: The efficacy and safety of continuous theta burst stimulation for auditory hallucinations: a systematic review and meta-analysis of randomized controlled trials
Source: Front Psychiatry. 2024 Aug 19;15:1446849. doi: 10.3389/fpsyt.2024.1446849 (PMC11366629; doi:10.3389/fpsyt.2024.1446849)
Supplement: Supplementary file 1 [file Table1.docx]

Supplementary Material

The efficacy and safety of continuous theta burst stimulation for auditory hallucinations: a systematic review and meta-analysis of randomized controlled trials

Shi-Yi Ye^1,2,†^, Chun-Nuan Chen^3,†^, Bo Wei^1,4,†^, Jin-Qiong Zhan^1,4^, Yi-Heng Li^1,4^, Chen Zhang^5,*^, Jing-Jing Huang^6,*^, Yuan-Jian Yang^1,4,*^

*** Correspondence:** Yuan-Jian Yang^*^: yuanjimyang@yeah.net; Jing-Jing Huang^*^: jjhuang_att@163.com; Chen Zhang^*^: [zhangchen645@gmail.com](mailto:zhangchen645@gmail.com)

**1.The PROSPERO register.**


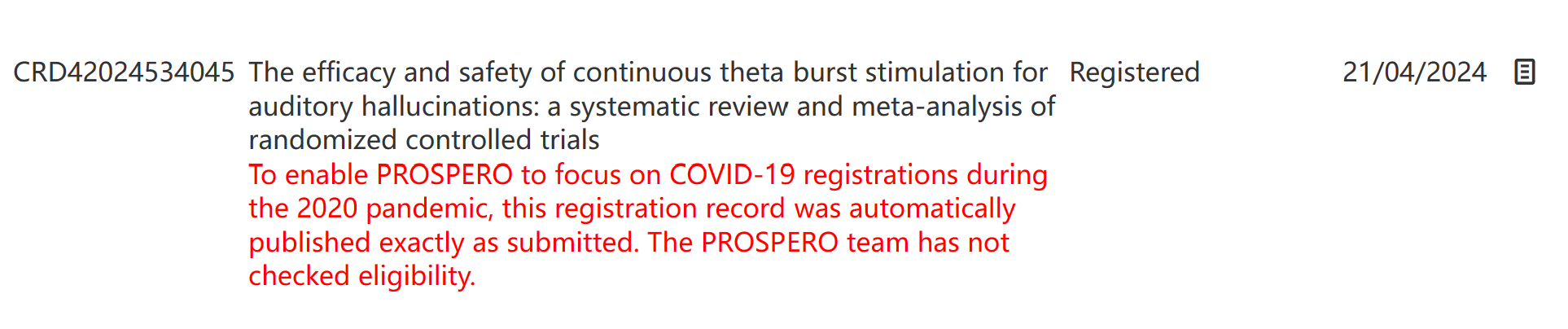


**2.Supplemental Table 1. Risk of bias summary**

|  | ***Randomization process*** | ***Deviations from intended interventions*** | ***Missing outcome data*** | ***Measurement of the outcome*** | ***Selection of the reported result*** | ***Overall*** |
| --- | --- | --- | --- | --- | --- | --- |
| Plewnia et al., 2014 | **+** | **+** | **+** | **+** | **+** | **+** |
| Koops et al., 2016 | **+** | **+** | **+** | **+** | **+** | **+** |
| Priya et al., 2022 | **+** | **+** | **+** | **+** | **+** | **+** |
| Liu et al., 2023 | **+** | **+** | **+** | **+** | **+** | **?** |

**+** : Low risk of bias; - : High risk of bias; ? : some concerns

1. **Supplemental Table 2. GRADE Analyses: cTBS for auditory hallucinations**

| **Primary/***secondary* outcomes | **Study (subjects)** | **Risk of bias** | **Inconsistency** | **Indirectness** | **Imprecision** | **Publication bias** | **Large effect** | **Overall quality of evidence^a^** |
| --- | --- | --- | --- | --- | --- | --- | --- | --- |
| ***The improvement of auditory hallucination at post-cTBS*** | 4 (151) | No | No | No | Serious^d^ | Undetected | No | +/+/+/-/; moderate |
| *Headache* | 2 (66) | No | No | No | Serious^d^ | Undetected | No | +/+/+/-/; moderate |
| *Pains* | 2 (80) | No | No | No | Serious^d^ | Undetected | No | +/+/+/-/; moderate |
| *Discontinuation due to any reason* | 3 (146) | No | No | No | Serious^d^ | Undetected | No | +/+/+/-/; moderate |
| ^a^GRADE Working Group grades of evidence: High quality=further research is very unlikely to change our confidence in the estimate of effect. Moderate quality=further research is likely to have an important impact on our confidence in the estimate of effect and could change the estimate. Low quality=further research is very likely to have an important impact on our confidence in the estimate of effect and is likely to change the estimate. Very low quality=we are very uncertain about the estimate.  ^b^Bias in randomization, bias in deviation from established interventions, bias in outcome measurements, bias in missing outcome data, and bias in selective reporting of outcomes  ^c^Meta-analytic results presented a serious inconsistency when I^2^ values were greater than 50% or P<0.1 in the Q statistics.  ^d^For continuous outcomes, N<400. For dichotomous outcomes, N<300.  ^e^Studies with large effects provided increased quality of evidence. Large effects=standard mean differences<-0.8 or >0.8 and risk ratios<0.5 or >2. | | | | | | | | |
